# The impact of confinement in the psychosocial behaviour due COVID-19 among members of a Brazilian university

**DOI:** 10.1177/0020764020971318

**Published:** 2020-11-08

**Authors:** Heloísa Monteiro Amaral-Prado, Filipy Borghi, Tânia Maron Vichi Freire Mello, Dora Maria Grassi-Kassisse

**Affiliations:** 1 LABEEST – Laboratory of Stress Study, Department of Structural and Functional Biology, Institute of Biology, University of Campinas – UNICAMP, Campinas, São Paulo, Brazil; 2 Department of Medical Psychology and Psychiatry, School of Medical Sciences, University of Campinas – UNICAMP, Campinas, São Paulo, Brazil

**Keywords:** Mental health, university, coping strategies, perceived stress, resilience, depression

## Abstract

**Background:**

The current situation due COVID-19 may cause an eminent impact on mental health because the confinement restrictions.

**Aims:**

The aim of this study was to analyze and compare perceived stress, resilience, depression symptoms and coping strategies on the members of University of Campinas, in Brazil, before and during the outbreak of the COVID-19.

**Methods:**

Volunteers over 18 years of both sexes, members of the University of Campinas (Unicamp) in Brazil answered instruments related to perceived stress, depression, resilience and coping strategies during final exams at the end of semester during 2018 to 2020.

**Results:**

We obtained 1,135 responses (893 before COVID-19 and 242 during COVID-19). The volunteers did not show significant differences for perceived stress, depressive signs and resilience before and during the pandemic. In both periods, men exhibited lower scores for perceived stress and depression and higher scores for resilience when compared to women. Undergraduate and graduate students exhibited higher perceived stress scores, more pronounced depressive signs and lower resilience, and employees and professors presented lower scores for perceived stress, depressive signs and greater resilience.

**Conclusions:**

These first months of confinement did not directly affect the scores of perceived stress, depression and resilience, however, each subgroup adapted to the new routine by changing the coping strategy used. This study suggests the importance of monitoring the mental health of member in the university, especially in times of epidemic, in the search for policies that aim to improve the resilience of the population and seek positive and effective coping strategies within the university environment.

## Introduction

The coronavirus disease 2019 (COVID-19) was firstly detected in Wuhan (Hubei Province, China) in December 2019 (Odriozola-González et al., 2020). However, the pandemic reached Latin America later than other continents (Lancet, 2020). Until 29th October 2020, Brazil has recorded 158,969 deaths and 5,494,376 confirmed cases of COVID-19 since the beginning of the pandemic, according to the National Council of Health Secretaries (Conass). This panorama confirms Brazil as the second country in the world with the highest number of cases and deaths in the pandemic of the new coronavirus.

In the absence of a vaccine, several countries have implemented a series of interventions to reduce contagious and decelerate progression of the pandemic (Aquino et al., 2020). One of the containment measures was the total confinement of the population in their homes, also known as lockdown, which led to the disruption of most daily activities (Ruiz-Roso et al., 2020). However, no national lockdown has been established in Brazil, but some communities and urban areas did declare a mandatory quarantine at different times. Confinement influences lifestyle and may induced many psychiatric individual and collective problems such as panic, anxiety, depression, post-traumatic stress disorders, among others symptoms (Jakovljevic et al., 2020). Efforts to reduce the spread of the COVID-19 virus among the younger and adult populations has prompted the suspensions of activities of schools, colleges, universities and other educational institutions in many countries (Sahu, 2020). The sudden change in the university’s routine generated several new demands, such as the use of technological resources that had never been used before to carry out classes and administrative activities, as well as remote work and/or study.

The university environment exposes its population to many situations linked to psychological distress (Byrd & McKinney, 2012; Pidgeon et al., 2014). The enhancement of resilience and coping strategies become even more important in this pandemic moment for better management of daily adversities, preventing the emergence of physical and mental health problems in the future, such as depression (Lupe et al., 2020; Vinkers et al., 2020). The current situation may cause an eminent impact on perceived stress, resilience, depression and coping strategies in this population due to the isolation restrictions, since all these topics are correlates (Mahmoud et al., 2012; Moreno-Fernandez et al., 2018). Given the expected impact of the situation due the confinement and COVID-19 crisis, the aim of this study was to analyze and compare perceived stress, resilience, depression symptoms and coping strategies on the members of University of Campinas, in Brazil, before and during the outbreak of the COVID-19.

## Methods

### Subjects

Volunteers over 18 years of both sexes, members of the University of Campinas (Unicamp) in Brazil were invited through the communications channels of the University and social media. The volunteers signed the free and informed consent, declaring understanding on the procedures that would be performed during the protocol. The study was conducted according to the guidelines laid down in the Declaration of Helsinki and ethical approval was granted from Research Ethics Committee of the School of Medical Sciences/University of Campinas (CAAE: 97370018.0.0000.5404). The volunteers were divided into four categories: undergraduate, graduate students (Master and PhD), employees and professors. All data were collected during the final exam/test month at the end of the semester (June and December) in two different periods: before COVID-19 (2018-2019) and during the outbreak of the COVID-19 (2020).

### Instruments

The participants answered five questionnaires that were validate for Portuguese language. The first questionnaire was a sociodemographic questionnaire to identify the volunteer’s sex, age, grade and period. The Perceived Stress Scale from Sheldon Cohen is the most widely used psychological instrument for measuring the perception of stress (Cohen et al., 1983). It is a measure of the degree to which situations in one’s life are appraised as stressful. Items were designed to assess how unpredictable, uncontrollable, and overloaded respondents find their lives to be. We used the Brazilian version with 14-question version, which total score is 56, with higher scores indicating a greater level of stress (Luft et al., 2007). The Patient Health Questionnaire-9 (PHQ-9) is a nine item questionnaire designed to screen for depression in primary care and other medical settings (Levis et al., 2019). The total score ranges from 0 to 27, which the standard cut-off score for screening to identify possible major depression is 10 or above (Arrieta et al., 2017). The Connor-Davidson Resilience Scale (CD-RISC10) is a questionnaire based on Connor and Davidson’s operational definition of resilience, which is the ability to ‘thrive in the face of adversity’. Since its development in 2003, the CD-RISC has been tested in a several contexts with a variety of populations. This work propose to work with the validated 10-item version of the measure validated for Portuguese language, called the CD-RISC10Brasil, which total score is 40, with higher scores indicating a greater resilience (Lopes & Martins, 2011). The Folkman and Lazarus Inventory of Coping Strategies includes the thoughts and actions people used to handle the internal or external demands of a specific stressful event. It is a list of 66 items answered using a Likert-type scale and the items are split into eight factors: confront, distance, self-control, social support, acceptance of responsibilities, escape-avoidance, problem solving and positive reappraisal (Folkman et al., 1986; Pompeo et al., 2016). This scale is not associated with a total score as a sum for assessment, as the items should be assessed using relative scores within each factor (Pompeo et al., 2016; Vitaliano et al., 1987). All data were collected and managed using REDCap^©^ electronic data capture tools hosted at Unicamp.

### Statistical analysis

Data are presented as means ± SEM. The normality was confirmed by D’Agostino-Pearson test. For parametric intragroup and intergroups comparisons, we performed One-way ANOVA followed by Tukey. For non-parametric data, we performed Friedman followed by Dunn’s for intragroup analysis and Kruskal–Wallis followed by Dunn’s for intergroups analysis. Two-tailed Pearson’s partial correlation for parametric data and Spearman’s partial correlation for non-parametric data was performed for correlations. All statistical analysis was done with Graph Pad Prism version 8.00 (Graph Pad Software, San Diego, California, USA). The acceptance level of significance was set at *p* < 0.05.

## Results

This study obtained 1135 responses from volunteers’ members from the University of Campinas (Unicamp). When separated by periods, 893 volunteers enrolled the surveys before the pandemic and 242 volunteers enrolled the surveys during the outbreak of the COVID-19. Demographic data showed a greater participation of women and individuals aged between 18 and 34 years. Considering the University situation, undergraduates were the one that most adhered to participate in the research, followed by graduate students, employees and professors (Table 1).

**Table 1.** Characteristics of the volunteers from the University of Campinas.

Characteristics	Number (%)	
	Before COVID-19	During COVID-19
Total	893 (100)	242 (100)
Sex		
Male	231 (25.8)	82 (33.8)
Female	653 (73.1)	156 (64.4)
Other	9 (1.1)	4 (1.8)
Age		
18–21	257 (28.7)	55 (22.8)
22–34	457 (51.1)	114 (47.1)
35–44	95 (10.6)	33 (13.6)
45–65	83 (9.2)	39 (16.1)
65+	1 (0.4)	1 (0.4)
University situation		
Undergraduate	460 (51.5)	104 (43.0)
Graduate students	194 (21.7)	59 (24.3)
Employees	190 (21.3)	50 (20.7)
Professors	49 (5.5)	29 (12.0)

The University members of this study did not show significant differences between the scores for perceived stress, depressive signs and resilience for the periods before and during the outbreak of the COVID-19. In both periods, men exhibited lower scores for perceived stress and depression and higher scores for resilience when compared to women. Volunteers who chose not to identify themselves as men or women were placed in a category named other. In the period before COVID-19, this category showed higher scores of perceived stress and depressive signs when compared to men. Compared to women, this category presented only higher scores for depressive signs. However, during the COVID-19 period, these differences were not observed (Table 2).

**Table 2.** Results of questionnaires according to sex and university situation of members from University of Campinas (mean ± SEM).

Sociodemographic variables	Mean scores					
	Sheldon Cohen	PHQ-9	CD-RISC10	Sheldon Cohen	PHQ-9	CD-RISC10
	Before COVID-19			During COVID-19		
Total	33.73 ± 0.30	12.09 ± 0.22	21.21 ± 0.25	33.94 ± 0.61	11.92 ± 0.41	21.90 ± 0.51
Sex						
Male	30.69 ± 0.62	10.16 ± 0.42	23.53 ± 0.49	29.79 ± 1.08	9.74 ± 0.69	25.11 ± 0.83
Female	34.71 ± 0.34*	12.68 ± 0.26*	20.42 ± 0.29*	36.06 ± 0.71*	12.99 ± 0.55*	20.25 ± 0.62*
Other	40.78 ± 2.06^#^	18.78 ± 2.05^# $^	19.11 ± 1.64	36.25 ± 2.62	14.50 ± 1.19	20.50 ± 2.63
University situation						
Undergraduate	36.10 ± 0.38	13.66 ± 0.30	19.70 ± 0.33	35.88 ± 0.99	13.09 ± 0.65	20.73 ± 0.78
Graduate students	34.04 ± 0.65	12.14 ± 0.46	21.20 ± 0.54	35.49 ± 1.03	13.05 ± 0.78	20.49 ± 0.96
Employees	29.42 ± 0.65^@ &^	9.46 ± 0.46^@ &^	23.76 ± 0.54^@ &^	30.58 ± 1.26^@ &^	10.04 ± 1.01^@^	24.14 ± 1.30
Professors	27.00 ± 1.29^@ &^	7.26 ± 0.90^@ &^	25.57 ± 1.01^@ &^	29.59 ± 1.73^@ &^	8.65 ± 1.31^@ &^	25.10 ± 0.87^@ &^

*Note.* ^*^p < 0.05 Male versus Female in the same period. ^#^*p* < 0.05 Male versus others in the same period. ^$^*p* < 0.05 Female versus others in the same period. ^@^*p* < 0.05 Undergraduate versus other university situations. ^&^*p* < 0.05 Graduate students versus other university situations.

Before COVID-19 period, undergraduate exhibited higher scores for perceived stress and depressive signs and lower resilience scores when compared to employees and professors. This behaviour was also observed during the epidemic, but the difference in the resilience score when compared to employees ceased to exist. Graduate students exhibited higher scores for perceived stress and depressive signs and lower resilience scores in the period before COVID-19 when compared to employees and professors. However, during the COVID-19 period, the difference between them and employees was no longer observed when the depressive signs and resilience scores were compared. Employees and professors did not exhibit differences in perceived stress, depressive signs and resilience between them in any period (Table 2).

For both periods analyzed, lower resilience scores lead to higher perceived stress and more accentuated depressive signs, as well as the greater resilience leads to an attenuation of the depressive signs, regardless of sex, university situation and period analyzed (Table 3).

**Table 3.** Correlation between perceived stress, depression and resilience according to sex and university situation of members from University of Campinas.

Sociodemographic variables	Correlation (r)					
	Sheldon Cohen vs. CD-RISC 10	Sheldon Cohen vs. PHQ-9	CD-RISC10 vs. PHQ-9	Sheldon Cohen vs. CD-RISC10	Sheldon Cohen vs. PHQ-9	CD-RISC10 vs. PHQ-9
	Before COVID-19			During COVID-19		
Total	−0.631*	0.761*	−0.511*	−0.667*	0.754*	−0.507*
Sex						
Male	−0.659*	0.799*	−0.568*	−0.647*	0.724*	−0.418*
Female	−0.609*	0.739*	−0.463*	−0.620*	0.714*	−0.473*
University situation						
Undergraduate	−0.587*	0.731*	−0.462*	−0.696*	0.742*	−0.532*
Graduate student	−0.588*	0.749*	−0.378*	−0.563*	0.699*	−0.393*
Employee	−0.638*	0.777*	−0.587*	−0.690*	0.768*	−0.489*
Professor	−0.601*	0.709*	−0.512*	−0.581*	0.773*	−0.415*

* *p* < 0.05.

Before the COVID-19 scenario, the coping strategy most often used by undergraduates was acceptance of responsibilities, and the least used was confront. For graduate students, the coping strategy most often used was social support, and the least used was confront. For employees and professors, the coping strategy most often used was problem solving, however, the least used strategy for employees was confront and for professors was escape-avoidance. During the epidemic, almost all groups presented new coping strategies. In the confinement period, undergraduates and graduate students started to choose the self-control as the lead coping strategy. As the least used strategy, undergraduates opted for positive reappraisal and graduate students for confront. Employees showed no changes in the most often and least used coping strategies in this new scenario. Professors started to use problem solving as the most often coping strategy but kept escape-avoidance as the less used strategy (Table 4).

**Table 4.** The Folkman and Lazarus Inventory of Coping Strategies according to university situation of members from University of Campinas (mean ± SEM).

	Coping strategies	Scores			
		Undergraduate	Graduate students	Employees	Professors
Before COVID-19	Confront	0.84 ± 0.02	0.80 ± 0.03	0.77 ± 0.03	0.70 ± 0.07
	Distance	1.01 ± 0.02	0.86 ± 0.03	0.84 ± 0.03	0.67 ± 0.05
	Self-control	1.33 ± 0.02	1.26 ± 0.04	1.26 ± 0.03	1.25 ± 0.08
	Social support	1.26 ± 0.03	1.45 ± 0.05	1.26 ± 0.04	1.15 ± 0.10
	Acceptance of responsibilities	1.51 ± 0.03	1.40 ± 0.05	1.14 ± 0.05	1.00 ± 0.10
	Escape-avoidance	1.32 ± 0.02	1.11 ± 0.04	0.86 ± 0.04	0.59 ± 0.07
	Problem solving	1.12 ± 0.02	1.26 ± 0.05	1.28 ± 0.04	1.33 ± 0.09
	Positive reappraisal	1.09 ± 0.02	0.94 ± 0.04	0.88 ± 0.03	0.79 ± 0.07
During COVID-19	Confront	0.94 ± 0.05	0.76 ± 0.07	0.81 ± 0.07	0.79 ± 0.09
	Distance	1.06 ± 0.06	0.90 ± 0.07	0.92 ± 0.08	0.76 ± 0.09
	Self-control	1.39 ± 0.05	1.25 ± 0.07	1.21 ± 0.07	1.18 ± 0.09
	Social support	1.28 ± 0.07	1.22 ± 0.09	1.20 ± 0.08	1.15 ± 0.13
	Acceptance of responsibilities	1.36 ± 0.07	1.12 ± 0.09	1.03 ± 0.10	1.00 ± 0.12
	Escape-avoidance	1.33 ± 0.06	1.13 ± 0.07	0.87 ± 0.09	0.73 ± 0.12
	Problem solving	1.13 ± 0.05	1.01 ± 0.08	1.30 ± 0.09	1.25 ± 0.10
	Positive reappraisal	0.92 ± 0.06	0.82 ± 0.07	1.03 ± 0.07	0.97 ± 0.11

As a next step, we performed correlations between the most used coping strategies in each group with the scores obtained in the other psychosocial instruments. In times before the pandemic, undergraduates exhibited a positive correlation with stress and depressive signs. Thus, there is a trend towards higher scores of perceived stress and depressive signs with greater use of accepting responsibility as a coping strategy in this group. Graduate students did not show any significant correlation with the psychosocial instruments used in this study. Employees and professors showed a negative correlation for perceived stress and a positive correlation for resilience. However, only employees showed a negative correlation between coping strategy and PHQ-9. Contrary to undergraduate students, the use of problem solving as a coping strategy leads to lower scores of perceived stress, depressive signs and greater resilience. During the pandemic, undergraduate and graduate students did not show significant correlations. Employees and professors maintained the same coping behaviour observed before the epidemic (Table 5).

**Table 5.** Correlation between coping strategies and psychometric instruments according to university situation of members from University of Campinas (mean ± SEM).

	University situation (coping strategy)	Correlation (r)		
		Sheldon Cohen versus coping strategy	PHQ-9 versus coping strategy	CD-RISC10 versus coping strategy
Before COVID-19	Undergraduate (Acceptance of responsibilities)	0.175^*^	0.291^*^	−0.057
	Graduate students (Social support)	0.036	0.043	0.106
	Employees (Problem solving)	−0.354^*^	−0.327^*^	0.448^*^
	Professors (Problem solving)	−0.479^*^	−0.249	0.499^*^
During COVID-19	Undergraduate (Self-control)	0.153	0.188	0.100
	Graduate students (Self-control)	−0.035	0.194	0.109
	Employees (Problem solving)	−0.463^*^	−0.349^*^	0.620^*^
	Professors (Problem solving)	−0.370^*^	−0.251	0.399^*^

* *p* < 0.05.

## Discussion

The ongoing COVID-19 pandemic forced the world to take protective measures such as lockdown of cities, travel bans, confinement and social distancing (Jakovljevic et al., 2020). However, literature suggests that restrictive measures such as quarantine, isolation and social distancing, have an impact on psychological wellbeing of people as well as emotive reactions to pandemic itself (Rubin & Wessely, 2020; Talevi et al., 2020). In this fashion, it was expected that the population into the university would be negatively impacted, increasing the stress experienced at the end of the semester. However, the scores for perceived stress, depression and resilience were not affected. What has changed is the way that each group deals with the new situation caused by COVID-19, highlighting the importance of the coping strategy as a quick response damper to keep the new routine without major losses.

Studies have documented differences between women and men with respect to symptom reporting, treatment seeking, coping strategies and several neurobiological variables pertinent to depression (Nayak et al., 2019; Wellman et al., 2018). It has been shown that women are twice as likely as men to develop depression and suffer stress, in addition to presenting less resilience (Nayak et al., 2019). The Unicamp community follows this same pattern described in the literature, with high scores for perceived stress, depressive signs and low score for resilience when compared to men. In our study, individuals who identified themselves with another option for sex or did not want to identify themselves with either sex exhibited a high score for perceived stress and depressive signs, and low scores for resilience. These data highlight the importance of further studies with this portion of the population for a better understanding of how to help deal with day-to-day problems. Discrimination with this minority of the population is still present today, which can be one of the aggravators for the highest scores in these individuals. Thus, the importance of inclusion measures becomes even more important.

The correlations observed before and during the COVID-19 pandemic in this study corroborate with studies from other countries, which also point to a positive correlation between perceived stress and depression and a negative correlation between stress and resilience (Kermott et al., 2019; Miceli et al., 2019). Positive coping strategies, which allow better coping with stress, enable the development of greater resilience, and consequently greater well-being for the individual. We observed that the average scores obtained in this study for perceived stress and depressive signs are higher the average of other populations studied (Ali et al., 2019; Polinski, 2020). High perceived stress can be associated with physical and psychological health problems, as well as with emotional intelligence and coping strategies employed (Enns et al., 2018).

In general, the Unicamp community showed different profiles among classified subpopulations, with undergraduate and graduate students with the lowest performance: higher perceived stress scores, more pronounced depressive signs and lower resilience. In the normal routine of the University, the undergraduates used acceptance of responsibilities as the main coping strategy, while graduate students used social support. During the pandemic, both groups started to use self-control as the main coping strategy. This change can be triggered by social isolation and by the transition from face-to-face teaching to online delivery. The use of self-control as the main coping strategy of this part of population becomes an important tool to face the new adversities, since the coping strategies previously used, in some way, have become limited due to the isolation predicted by the pandemic. However, regardless of the periods analyzed, these strategies were not shown to be effective enough for improvement or good management and coping with stressors.

Groups that have employment relationship with the University, such as employees and professors, showed the best performances: lower scores for perceived stress, depressive signs and greater resilience. These subgroups of the community used the problem solving as main coping strategy in both periods analyzed. The negative correlations between problem solving with perceived stress and depressive signs questionnaires, as well as the positive correlation between problem solving and resilience, indicate a better management of day-to-day stressors when these subgroups applied this strategy. Therefore, the coping strategy focused on the problem, seeking to change and resolve the conflict, such as problem solving, proved to be effective.

Professors were the group that had the best performance among all. Regardless of the period, they were the least to use escape-avoidance as a coping strategy when compared to other groups. This fact may indicate that this strategy may have a relationship with a possible increase in perceived stress and depressive signs, as well as less resilience, since the other subgroups showed greater use of this strategy compared to professors. The literature indicates escape-avoidance as a negative adaptation of human behaviour in dealing with stressful situations, which can lead to feelings of isolation and loneliness, and as a consequence the presence of apathy and demotivation to perform the activities that the individual needs to perform, such as example, academic or professional activities (Pruessner et al., 2020).

Studies in many populations have alarmed the impact of social isolation on mental health caused by COVID-19. Thus, the pandemic period stimulated many cross-sectional studies on mental health, mainly in groups without previous studies. These studies can often highlight findings due to the fact that there are no previous comparisons. Cross-sectional studies with health-care workers from UK during COVID-19 alarmed to the great incidence of mental health problems, however, longitudinal studies with prepandemic and postpandemic groups found no increase in mental distress among health-care workers due to COVID-19 compared with the general population (Lamb et al., 2020). Therefore, this type of information highlights the importance of longitudinal assessments that allows to track changes and better assess the situation without unnecessarily pathologizing common responses.

Despite the limitations, this is a cross-sectional study conducted at a Brazilian university that started before an unprecedented situation, which allowed a screening of the new situation within the university. This study was performed in only university from Brazil, which may contribute to some bias in the study results. The adherence from other universities in the same model of this study may extend and generalize the findings. Moreover, the present study utilized self-report measures that considered the framework of an individual’s subjective perception of perceived stress, depression and resilience.

## Conclusion

Regardless of the emerging context of COVID-19, the Unicamp community exhibited high scores for perceived stress and depressive signs and low scores for resilience when compared to studies around the world. This population have been specially impacted by the COVID-19 confinement, but, in these first months of confinement, it did not directly affect the scores of perceived stress, depression and resilience. On the other hand, each subgroup adapted to the new routine by changing the coping strategy used. Groups with lower performances, such as undergraduate and graduate students, opted for new strategies to face adversity, while groups with the best performances, such as employees and professor, already presented efficient strategies and did not feel obliged to discover new ways to face the new situation imposed by COVID-19. This study suggests the importance of monitoring the mental health of member in the university, especially in times of epidemic, in the search for policies that aim to improve the resilience of the population and seek positive and effective coping strategies within the university environment.

## References

[bibr1-0020764020971318] Ali N. M. Nowshad N. A. Mansoor K. M. Ibnouf R. A. Albehiery R. M. Carrick F. R. Abdulrahman M. (2019). Perceived academic and psychological stress among adolescents in United Arab Emirates: Role of gender, age, depression, and high expectation of parents. Psychiatria Danubina, 31(Suppl. 3), 331–337. 31488749

[bibr2-0020764020971318] Aquino E. M. L. Silveira I. H. Pescarini J. M. Aquino R. Souza-Filho J. A. Rocha A. S. Ferreira A. Victor A. Teixeira C. Machado D. B. Paixão E. Alves F. J. O. Pilecco F. Menezes G. Gabrielli L. Leite L. Almeida M. C. C. Ortelan N. Fernandes Q. H. R. F. Ortiz R. J. F. … Lima R. T. R. S . (2020). Social distancing measures to control the COVID-19 pandemic: Potential impacts and challenges in Brazil. Ciencia & Saude Coletiva, 25(Suppl. 1), 2423–2446. 10.1590/1413-81232020256.1.10502020 32520287

[bibr3-0020764020971318] Arrieta J. Aguerrebere M. Raviola G. Flores H. Elliott P. Espinosa A. Reyes A. Ortiz-Panozo E. Rodriguez-Gutierrez E. G. Mukherjee J. Palazuelos D. Franke M. F. (2017). Validity and Utility of the Patient Health Questionnaire (PHQ)-2 and PHQ-9 for screening and diagnosis of depression in Rural Chiapas, Mexico: A cross-sectional study. Journal of Clinical Psychology, 73(9), 1076–1090. 10.1002/jclp.22390 28195649 PMC5573982

[bibr4-0020764020971318] Byrd D. R. McKinney K. J. (2012). Individual, interpersonal, and institutional level factors associated with the mental health of college students. Journal of the American College Health Association, 60(3), 185–193. 10.1080/07448481.2011.584334 22420695

[bibr5-0020764020971318] Cohen S. Kamarck T. Mermelstein R. (1983). A global measure of perceived stress. Journal of Health and Social Behavior, 24(4), 385–396. 6668417

[bibr6-0020764020971318] Enns A. Eldridge G. D. Montgomery C. Gonzalez V. M. (2018). Perceived stress, coping strategies, and emotional intelligence: A cross-sectional study of university students in helping disciplines. Nurse Education Today, 68, 226–231. 10.1016/j.nedt.2018.06.012 30053557

[bibr7-0020764020971318] Folkman S. Lazarus R. S. Gruen R. J. DeLongis A. (1986). Appraisal, coping, health status, and psychological symptoms. Journal of Personality and Social Psychology, 50(3), 571–579. 3701593 10.1037//0022-3514.50.3.571

[bibr8-0020764020971318] Jakovljevic M. Bjedov S. Jaksic N. Jakovljevic I. (2020). COVID-19 pandemia and public and global mental health from the perspective of global health securit. Psychiatria Danubina, 32(1), 6–14. 10.24869/psyd.2020.6 32303023

[bibr9-0020764020971318] Kermott C. A. Johnson R. E. Sood R. Jenkins S. M. Sood A. (2019). Is higher resilience predictive of lower stress and better mental health among corporate executives? PLoS One, 14(6), e0218092. 10.1371/journal.pone.0218092 PMC6559706 31185049

[bibr10-0020764020971318] Lamb D. Greenberg N. Stevelink S. A. M. Wessely S. (2020). Mixed signals about the mental health of the NHS workforce. The Lancet Psychiatry. Advance online publication. 10.1016/S2215-0366(20)30379-5 PMC7470862 32891218

[bibr11-0020764020971318] Lancet T. (2020). COVID-19 in Brazil: “So what?”. Lancet (London, England), 395(10235), 1461. 10.1016/S0140-6736(20)31095-3 PMC7251993 32386576

[bibr12-0020764020971318] Levis B. Benedetti A. Thombs B. D. (2019). Accuracy of Patient Health Questionnaire-9 (PHQ-9) for screening to detect major depression: Individual participant data meta-analysis. BMJ (Clinical research ed.), 365, l1476. 10.1136/bmj.l1476 PMC6454318 30967483

[bibr13-0020764020971318] Lopes V. R. Martins M. D. C. F . (2011). Factorial validation and adaptation of the connor-davidson resilience scale (CD-RISC-10) for brazilians. Revista Psicologia Organizações e Trabalho, 11(2), 36–50.

[bibr14-0020764020971318] Luft C. D. B. Sanches S. D. O. Mazo G. Z. Andrade A. (2007). Brazilian version of the perceived stress scale: Translation and validation for the elderly. Revista de Saúde Pública, 41(4), 606–615. 10.1590/S0034-89102007000400015 17589759

[bibr15-0020764020971318] Lupe S. E. Keefer L. Szigethy E. (2020). Gaining resilience and reducing stress in the age of COVID-19. Current Opinion in Gastroenterology, 36(4), 295–303. 10.1097/MOG.0000000000000646 32398567

[bibr16-0020764020971318] Mahmoud J. S. R. Staten R. Hall L. A. Lennie T. A. (2012). The relationship among young adult college students’ depression, anxiety, stress, demographics, life satisfaction, and coping styles. Issues in Mental Health Nursing, 33(3), 149–156. 10.3109/01612840.2011.632708 22364426

[bibr17-0020764020971318] Miceli J. Geller D. Tsung A. Hecht C. L. Wang Y. Pathak R. Cheng H. Marsh W. Antoni M. Penedo F. Burke L. Ell K. Shen S. Steel J. (2019). Illness perceptions and perceived stress in patients with advanced gastrointestinal cancer. Psycho-Oncology, 28(7), 1513–1519. 10.1002/pon.5108 31090125 PMC6610754

[bibr18-0020764020971318] Moreno-Fernandez R. D. Tabbai S. Castilla-Ortega E. Perez-Martin M. Estivill-Torrus G. Rodriguez de Fonseca F. Santin L.J. Pedraza C. (2018). Stress, depression, resilience and ageing: A role for the LPA-LPA1 pathway. Current Neuropharmacology, 16(3), 271–283. 10.2174/1570159X15666170710200352 28699486 PMC5843979

[bibr19-0020764020971318] Nayak A. S. Parkar S. R. Nachane H. B. Sangoi B. A. Shinde R. G. (2019). Gender variability of perceived stress and negative inferential feedback in depression. Indian Journal of Psychological Medicine, 41(4), 331–337. 10.4103/IJPSYM.IJPSYM_343_18 31391665 PMC6657475

[bibr20-0020764020971318] Odriozola-González P. Planchuelo-Gómez Á. Irurtia M. J. de Luis-García R. (2020). Psychological effects of the COVID-19 outbreak and lockdown among students and workers of a Spanish university. Psychiatry Research, 290, 113108. 10.1016/j.psychres.2020.113108 32450409 PMC7236679

[bibr21-0020764020971318] Pidgeon A. M. Rowe N. F. Stapleton P. Magyar H. B. Lo B. C. Y. (2014). Examining characteristics of resilience among university students: An international study. Open Journal of Social Sciences, 2(11), 14–22. 10.4236/jss.2014.211003

[bibr22-0020764020971318] Polinski K. J. Bemis E. A. Feser M. Seifert J. Demoruelle M. K. Striebich C. C. Brake S. O’Dell J. R. Mikuls T. R. Weisman M. H. Gregersen P. K. Keating R. M. Buckner J. Nicassio P. Holers V. M. Deane K. D. Norris J. M. (2020). Perceived stress and inflammatory arthritis: A prospective investigation in the studies of the etiologies of rheumatoid arthritis (SERA) cohort. Arthritis Care & Research. Advance online publication. 10.1002/acr.24085 PMC7145743 31600025

[bibr23-0020764020971318] Pompeo D. A. Carvalho A. D. Olive A. M. Souza M. D. G. Galera S. A. (2016). Strategies for coping with family members of patients with mental disorders. Revista Latino-Americana de Enfermagem, 24, e2799. 10.1590/1518-8345.1311.2799 27627121 PMC5048725

[bibr24-0020764020971318] Pruessner L. Barnow S. Holt D. V. Joormann J. Schulze K. (2020). A cognitive control framework for understanding emotion regulation flexibility. Emotion (Washington, D.C.), 20(1), 21–29. 10.1037/emo0000658 31961173

[bibr25-0020764020971318] Rubin G. J. Wessely S. (2020). The psychological effects of quarantining a city. BMJ (Clinical research ed.), 368, m313. 10.1136/bmj.m313 31992552

[bibr26-0020764020971318] Ruiz-Roso M. B. de Carvalho Padilha P. Mantilla-Escalante D. C. Ulloa N. Brun P. Acevedo-Correa D. Arantes Ferreira Peres W. Martorell M. Aires M. T. de Oliveira Cardoso L. Carrasco-Marín F. Paternina-Sierra K. Rodriguez-Meza J. E. Montero P. M. Bernabè G. Pauletto A. Taci X. Visioli F. Dávalos A. (2020). COVID-19 confinement and changes of adolescent’s dietary trends in Italy, Spain, Chile, Colombia and Brazil. Nutrients, 12(6), 1807. 10.3390/nu12061807 PMC7353171 32560550

[bibr27-0020764020971318] Sahu P. (2020). Closure of universities due to coronavirus disease 2019 (COVID-19): Impact on education and mental health of students and academic staff. Cureus, 12(4), e7541. 10.7759/cureus.7541 32377489 PMC7198094

[bibr28-0020764020971318] Talevi D. Socci V. Carai M. Carnaghi G. Faleri S. Trebbi E. di Bernardo A. Capelli F. Pacitti F. (2020). Mental health outcomes of the COVID-19 pandemic. Rivista di Psichiatria, 55(3), 137–144. 10.1708/3382.33569 32489190

[bibr29-0020764020971318] Vinkers C. H. van Amelsvoort T. Bisson J. I. Branchi I. Cryan J. F. Domschke K. Howes O. D. Manchia M. Pinto L. de Quervain D. Schmidt M. V. van der Wee N. J. A. (2020). Stress resilience during the coronavirus pandemic. European Neuropsychopharmacology: The Journal of the European College of Neuropsychopharmacology, 35, 12–16. 10.1016/j.euroneuro.2020.05.003 32446705 PMC7211573

[bibr30-0020764020971318] Vitaliano P. P. Maiuro R. D. Russo J. Becker J. (1987). Raw versus relative scores in the assessment of coping strategies. Journal of Behavioral Medicine, 10(1), 1–18. 10.1007/BF00845124 3585998

[bibr31-0020764020971318] Wellman C. L. Bangasser D. A. Bollinger J. L. Coutellier L. Logrip M. L. Moench K. M. Urban K. R. (2018). Sex differences in risk and resilience: Stress effects on the neural substrates of emotion and motivation. The Journal of Neuroscience: The Official Journal of the Society for Neuroscience, 38(44), 9423–9432. 10.1523/JNEUROSCI.1673-18.2018 30381434 PMC6209838

